# Development and validation of multivariable risk adjustment models for return of spontaneous circulation and survival to hospital discharge following out-of-hospital cardiac arrest in England

**DOI:** 10.1093/ehjqcco/qcaf159

**Published:** 2025-12-18

**Authors:** Adam J Boulton, Chen Ji, Gavin D Perkins, Terry P Brown, Joyce Yeung

**Affiliations:** Warwick Clinical Trials Unit, Warwick Medical School, University of Warwick, Coventry CV4 7AL, UK; Warwick Clinical Trials Unit, Warwick Medical School, University of Warwick, Coventry CV4 7AL, UK; Warwick Clinical Trials Unit, Warwick Medical School, University of Warwick, Coventry CV4 7AL, UK; Critical Care Unit, University Hospitals Birmingham NHS Foundation Trust, Birmingham B9 5SS, UK; Out-of-Hospital Cardiac Arrest Outcome (OHCAO) Project, Clinical Trials Unit, Warwick Medical School, University of Warwick, Coventry CV4 7AL, UK; Applied Research Collaboration West Midlands (ARCWM), Clinical Trials Unit, Warwick Medical School, University of Warwick, Coventry CV4 7AL, UK; Warwick Clinical Trials Unit, Warwick Medical School, University of Warwick, Coventry CV4 7AL, UK; Critical Care Unit, University Hospitals Birmingham NHS Foundation Trust, Birmingham B9 5SS, UK

**Keywords:** Emergency medical services, Cardiopulmonary resuscitation, Health services research, Registries, Benchmarking

## Abstract

**Aims:**

Risk adjustment models can support clinical decision-making and enable comparative reporting to drive quality improvement. To develop and validate risk adjustment models for return of spontaneous circulation (ROSC) at hospital handover and survival to hospital discharge among patients experiencing out-of-hospital cardiac arrest (OHCA), using national registry data.

**Methods and results:**

Patients with OHCA in England from 1 January 2016 and 31 December 2017, where resuscitation was attempted by the emergency medical service, were included. Data were sourced from the Out-of-Hospital Cardiac Arrest Outcomes registry. The 2016 cohort (*n* = 27 942) was used for model development and the 2017 cohort (*n* = 28 425) for validation. Outcomes were ROSC at hospital handover and survival to hospital discharge. Candidate predictors were age, sex, witnessed status, aetiology, bystander CPR, initial rhythm, and public access defibrillator use. Multivariable logistic regression models were developed using backward stepwise selection. Model performance was assessed using area under the receiver operating characteristic curve (AUC), Brier score, calibration plots, Hosmer–Lemeshow test, and classification metrics. Across the full study dataset, ROSC occurred in 28.6% of cases and survival in 8.2%. All candidate predictors were retained for ROSC, while sex was excluded from the final survival model. The ROSC model had an AUC of 0.702 (95% CI: 0.694–0.711) in development and 0.712 (0.704–0.719) in validation, with Brier scores of 0.182 in both. The survival model had an AUC of 0.877 (0.868–0.887) in development and 0.871 (0.862–0.879) in validation, with Brier scores of 0.059 and 0.061, respectively.

**Conclusion:**

These validated models demonstrated strong performance, improved on previous models, and may support benchmarking, audit, and quality improvement initiatives.

## Graphical Abstract

**Graphical Abstract:**
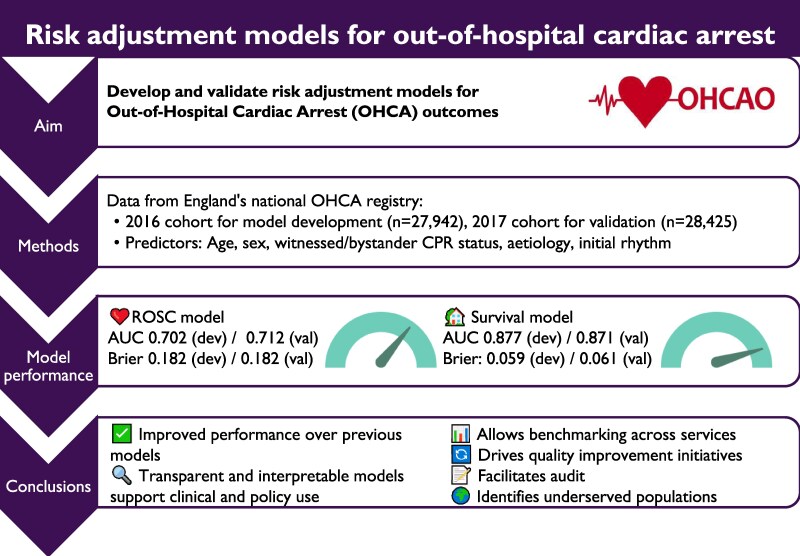

Key Learning Points **What is already known:**

- Risk adjustment models allow benchmarking, audit, and quality improvement in out-of-hospital cardiac arrest.
- Existing models have been limited by missing data and complex structures.

**What this study adds:**

- This study presents validated risk adjustment models for ROSC and survival using national registry data, with strong performance that improves upon previous models.
- The models demonstrate consistent discrimination and calibration across development and validation datasets, supporting their use in benchmarking and audit.
- A simplified model structure without interaction terms enhances clinical usability, as well as improving performance.

## Introduction

Out-of-hospital cardiac arrest (OHCA) is a leading cause of death and disability worldwide and is a significant concern for healthcare systems.^1-3^ In England, emergency medical services (EMS) attempt resuscitation in around 35 000 OHCA patients each year.^4^ Despite advances in EMS care and public health initiatives to improve rates of bystander cardiopulmonary resuscitation (CPR) and public access defibrillator availability and use, survival remains poor, with fewer than one in ten patients surviving to leave hospital.^2,4^

Variation exists in the care provided for OHCA patients.^5-10^ Risk adjustment models can enable fair comparisons across services and populations by accounting for differences in case mix, thereby supporting benchmarking, audit, and targeted quality improvement. They are widely used in other clinical contexts, including in-hospital cardiac arrest and intensive care, to guide service evaluation and improvement.^11-13^ Disparities exist for OHCA patients, particularly in incidence rates and provision of bystander CPR, reflecting broader health inequalities associated with ethnicity and socio-economic status.^14-19^ Accurate risk adjustment can also help identify disadvantaged populations and underserved areas, facilitating equitable improvements in care. As patient populations change and clinical practice advances, periodic updates of models are needed to maintain their relevance and accuracy.^20-22^ National cardiac arrest registries offer valuable opportunities to better understand the epidemiology and outcomes of OHCA. In England, the Out-of-Hospital Cardiac Arrest Outcomes (OHCAO) registry captures detailed, individual-level data on EMS-treated OHCAs, aligned with the Utstein template.^23,24^ While previous studies have developed risk adjustment models using this registry, they were limited by missing data.^25^ A model with a simpler structure without interaction terms may also benefit interpretation, provided performance is maintained.^26^ With continual improvements in data completeness and quality, as well as improvements in the community response and treatment of OHCA, there is a timely need to update and refine these models. The aim of this study was to develop and validate risk adjustment models for return of spontaneous circulation (ROSC) at hospital handover and survival to hospital discharge following out-of-hospital cardiac arrest, using a national registry.

## Methods

The Transparent Reporting of a multivariable prediction models for Individual Prognosis Or Diagnosis (TRIPOD) statement was followed in the reporting of this study.^27^ This study is part of a wider research project that received Health Research Authority and Research Ethics Committee approval (23/NW/0378). There was a pre-specified protocol and prospective registration (researchregistry10307).^28^

### Data source

Data were obtained from the OHCAO registry held at the University of Warwick. This national registry collects comprehensive, individual-level case information from all National Health Service ambulance services in England for patients suffering an OHCA where resuscitation is initiated or continued by EMS personnel.^3,23^ Routinely collected variables are informed by the Utstein template and include patient variables, process variables, and outcome variables.^24^ The OHCAO registry has embedded quality assurance processes to ensure accuracy of collected data, including automated audits and site data queries. The study population included was all OHCA cases within the registry between 1 January 2016 and 31 December 2017. Data from 2016 were used for model development, and data from 2017 were used for model validation.

### Variables

The outcomes of interest were survival and ROSC. Survival was measured at the point of hospital discharge and ROSC was measured at the point of EMS handover to hospital. Candidate predictor variables were informed by previous research and expert clinician knowledge. These included pre-EMS intervention variables, which were age, sex, witnessed status, aetiology of arrest, bystander CPR, initial rhythm, and use of a public access defibrillator by the public.^25,29^ Age was included as a continuous variable, and all other variables were categorical. Within aetiology, traumatic and exsanguination were combined, whilst all other categories were used unprocessed from the registry. A sensitivity analysis was conducted where asphyxia, drowning, and overdose were combined into a single category, consistent with previous models using OHCAO registry data.^25^ Witnessed status and bystander CPR were combined into one variable. Within the registry dataset, EMS witnessed cases are considered as not receiving bystander CPR. This risks biasing the assessment of bystander CPR since the EMS witnessed cases typically differ in time to start of resuscitation and time to receipt of advanced interventions, all of which influence outcome.^30^ These variables were recoded to produce categories of unwitnessed and no bystander CPR; unwitnessed and bystander CPR provided; bystander witnessed and no bystander CPR; bystander witnessed and bystander CPR provided; and EMS witnessed. Initial rhythm was recoded into clinically meaningful categories: shockable (ventricular fibrillation or pulseless ventricular tachycardia); pulseless electrical activity; and asystole. EMS response time and service were not included as they reflect system-level performance rather than pre-EMS patient-level characteristics. The intended application of these models was for comparative performance and quality improvement across systems, and hence predictors were restricted to patient-related factors. Complete cases for each outcome were used. Missingness for each variable was examined and reported for the full dataset. There was high missingness for public access defibrillator use and therefore this variable was used in sensitivity analysis only. Due to the potential heterogeneity of the missingness level and missing mechanisms across ambulance services, no imputation was conducted for this variable.

### Analytical methods

Variables were summarized using descriptive statistics. Age was determined to be non-normally distributed after inspection of Q–Q plot and histogram; therefore, median and interquartile range were used. Logistic regression models were developed separately for each outcome of interest. First, univariable models were constructed for all candidate predictor variables to identify key associations. Second, variables with significant univariable association (*P* < 0.05) were included in multivariable models, and a backward stepwise selection process was used to derive a parsimonious final model. This approach was pre-specified in the protocol and preferred over alternatives such as forward selection, bidirectional selection, and LASSO, as it allowed the model to begin with all clinically relevant variables followed by systematic removal to maintain interpretability and clinical relevance. All candidate predictors were included in the initial model, and variables sequentially removed if this resulted in a lower Akaike Information Criterion (AIC) to indicate improved model parsimony, provided that the overall model performance was not worsened. Logistic regression assumptions were checked prior to investigating model performance. Independence of observations was examined using a standardized residual against observation index plot, multicollinearity was assessed by calculating the variance inflation factor for each predictor, and linearity of the continuous predictor (age) with logit of the outcome was evaluated graphically.

Several sensitivity analyses were conducted to assess the robustness of risk adjustment models. Firstly, models were constructed including cases with information on public access defibrillator use. Secondly, potentially influential observations were identified using Cook’s distance, which combines residual and leverage information, and were excluded following visual inspection of diagnostic plots (see Supplementary Material). Thirdly, aetiology was recoded to match the categorization used in previous models, as described above. Fourthly, for survival models, sex was reintroduced after its exclusion by backward stepwise selection due to its clinical relevance and potential role as a co-variable. This analysis assessed whether excluding sex materially affected model estimates or predictive performance. Finally, multiple imputation using chain equations for missing data in the validation datasets. The model performance was summarized as pooled means and standard deviations across 20 imputed datasets.

Model performance was examined on the development and validation datasets using assessments pre-specified in the study protocol. This included a range of discrimination, calibration, and classification metrics. Discrimination was evaluated using receiver operating characteristic (ROC) curve plots and area under the curve (AUC) values, which reflect the model’s ability to distinguish between outcomes. The Brier score was used to quantify overall model performance, reflecting both calibration and discrimination, with lower scores indicating better performance. Calibration was assessed using calibration plots and the Hosmer–Lemeshow goodness-of-fit test, which measure how closely predicted probabilities align with observed outcomes. Classification accuracy was evaluated using the confusion matrix, using a predicted classification threshold of 0.5, from which sensitivity, specificity, positive predictive value, negative predictive value, and overall accuracy (proportion of correctly classified cases) were calculated. Model selection was guided by the AIC, which balances model fit and complexity. All data processing, analysis, and model construction were performed using R (version 4.5.1) with the following packages enabled: mice, pROC, probably, tidymodels, tidyverse.^31^ The R code used is available on reasonable request.

## Results

A total of 27 942 patients were included in the development dataset and 28 425 patients in the validation dataset. The characteristics and outcomes of these datasets are summarized in *Table 1*. Across both datasets, the rate of ROSC at hospital handover was 28.5%, and survival to hospital discharge was 8.2%. Data quality was similar between both datasets, and the pattern of missing data is described and visualized in Supplementary Material. The characteristics of incomplete, and therefore excluded cases, as well as complete cases are reported in the Supplementary Material.

**Table 1:** Characteristics of development and validation datasets

Variable	Development data (*n* = 27 942)	Validation data (*n* = 28 425)
Age—median (IQR)	71 (24)	71 (24)
Missing—*N* (%)	4042 (14.5%)	3923 (13.8%)
Sex—*N* (%)		
Male	17 551 (62.8%)	18 078 (63.6%)
Female	9925 (35.5%)	10 179 (35.8%)
Missing	466 (1.7%)	168 (0.6%)
Witnessed status—*N* (%)		
Unwitnessed	9512 (34.0%)	9178 (34.2%)
Bystander witnessed	11 350 (40.6%)	12 959 (45.6%)
EMS witnessed	3739 (13.4%)	3455 (12.2%)
Missing	3341 (12.0%)	2293 (8.1%)
Bystander CPR—N (%)		
Yes	15 358 (55.0%)	16 472 (57.9%)
No	10 006 (35.8%)	10 417 (36.6%)
NA (EMS witnessed)	358 (1.3%)	95 (0.3%)
Missing	2220 (7.9%)	1441 (5.1%)
Public access defibrillator—*N* (%)		
Yes	809 (2.9%)	894 (3.1%)
No	15 006 (53.7%)	15 076 (53.0%)
Missing	12 127 (43.4%)	12 455 (43.8%)
Aetiology—*N* (%)		
Medical	21 166 (75.7%)	22 668 (79.7%)
Asphyxia	604 (2.2%)	797 (2.8%)
Drowning	87 (0.3%)	90 (0.3%)
Overdose	428 (1.5%)	431 (1.5%)
Traumatic	713 (2.6%)	787 (2.8%)
Exsanguination	10 (0.04%)	13 (0.05%)
Other (non-cardiac)	2082 (7.5%)	1376 (4.8%)
Missing	2852 (10.2%)	2263 (8.0%)
Initial rhythm—*N* (%)		
Shockable (VF/VT)	5532 (19.8%)	5860 (20.6%)
Asystole	14 629 (52.4%)	14 818 (52.1%)
PEA	6007 (21.5%)	6155 (21.7%)
Missing	1774 (6.3%)	1592 (5.6%)
ROSC at hospital handover—*N* (%)		
Yes	7364 (26.4%)	7902 (27.8%)
No	19 019 (68.1%)	19 102 (67.2%)
Missing	1559 (5.6%)	1421 (5.0%)
Survival to hospital discharge—*N* (%)		
Yes	2039 (7.3%)	2285 (8.0%)
No	24 093 (86.2%)	24 365 (85.7%)
Missing	1810 (6.5%)	1775 (6.2%)

CPR, cardiopulmonary resuscitation; EMS, emergency medical services; IQR, interquartile range; PEA, pulseless electrical activity; ROSC, return of spontaneous circulation; VF, ventricular fibrillation; VT, ventricular tachycardia.

### Model development

Complete cases were used for model development, including 17 709 for the ROSC model and 17 546 for the survival to hospital discharge model. In univariable logistic regression analyses, all candidate predictor variables except age and sex were significantly associated with ROSC (*Table 2*). For survival, all candidate predictor variables were significantly associated with the outcome (*Table 2*).

**Table 2:** Univariable models odds ratios

Variable	ROSC at hospital handover odds ratio (95% CI)	Survival to hospital discharge odds ratio (95% CI)
Age	1.000 (0.999–1.002)	0.982 (0.979–0.984)
Sex		
Male	1	1
Female	1.066 (0.996–1.140)	0.644 (0.572–0.724)
Witness/bystander CPR		
Unwitnessed and no bystander CPR	1	1
Unwitnessed and bystander CPR provided	0.863 (0.758–0.985)	0.798 (0.596–1.074)
Bystander witnessed and no bystander CPR	1.685 (1.469–1.934)	1.913 (1.446–2.550)
Bystander witnessed and bystander CPR provided	2.168 (1.932–2.438)	3.438 (2.718–4.410)
EMS witnessed	2.473 (2.178–2.811)	5.946 (4.676–7.661)
Aetiology		
Medical	1	1
Asphyxia	1.120 (0.921–1.356)	0.349 (0.206–0.549)
Drowning	0.353 (0.156–0.698)	0.323 (0.053–1.032)
Overdose	0.957 (0.745–1.220)	1.226 (0.845–1.725)
Other (non-cardiac)	1.068 (0.954–1.194)	0.464 (0.361–0.587)
Traumatic and exsanguination	0.633 (0.497–0.798)	0.192 (0.087–0.362)
Initial rhythm		
Shockable (VF/VT)	1	1
Asystole	0.199 (0.183–0.216)	0.036 (0.030–0.043)
PEA	0.426 (0.390–0.466)	0.110 (0.094–0.129)
Public access defibrillator	(*n* = 15 638)	(*n* = 15 387)
No	1	1
Yes	1.468 (1.267–1.699)	1.920 (1.559–2.347)

CPR, cardiopulmonary resuscitation; EMS, emergency medical services; PEA, pulseless electrical activity; ROSC, return of spontaneous circulation; VF, ventricular fibrillation; VT, ventricular tachycardia.

**Figure 1:**
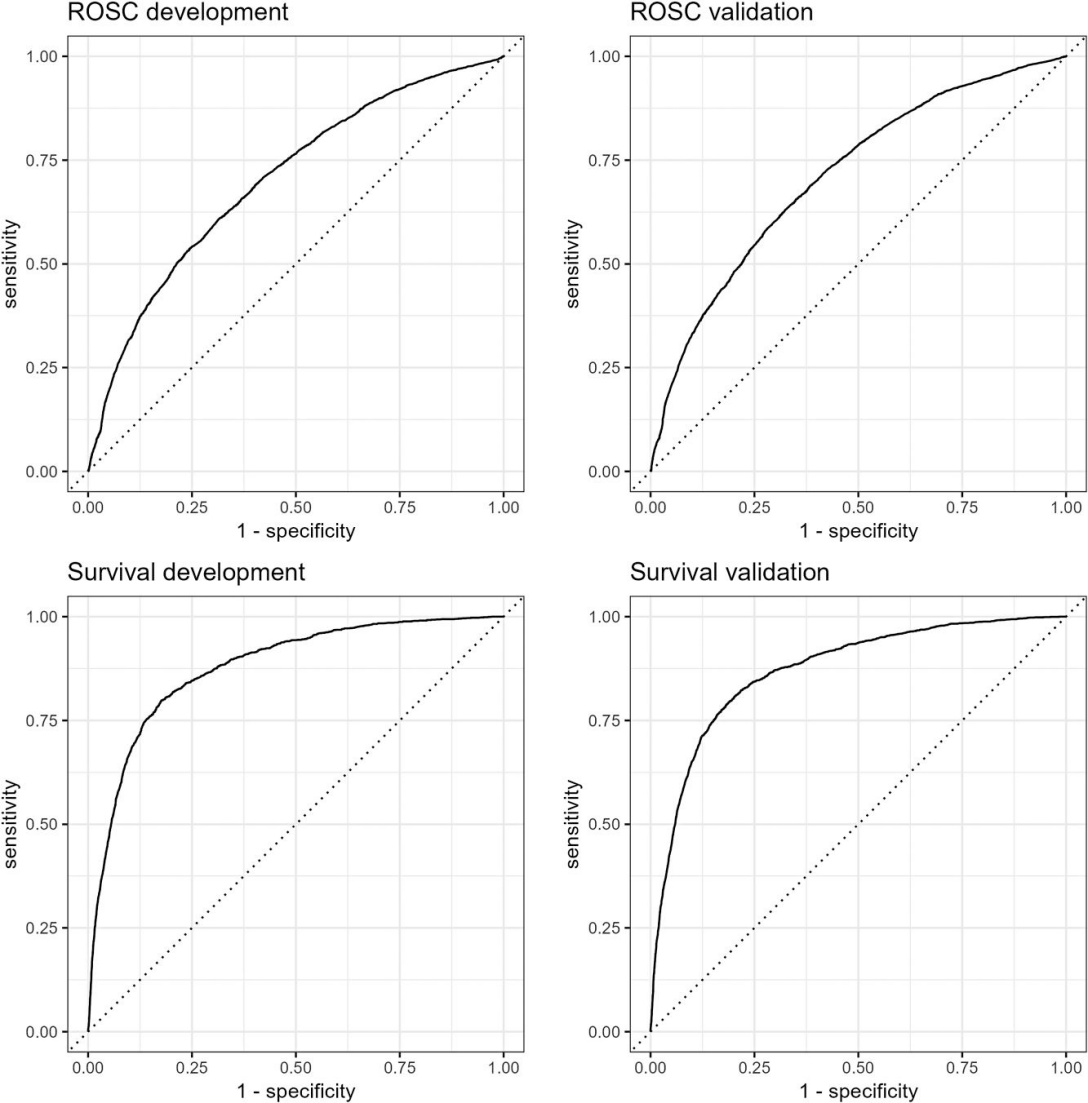
Receiver operating characteristic curves. ROSC: Return of spontaneous circulation.

The initial multivariable model of ROSC included all candidate predictor variables. During backward stepwise selection, removal of age marginally improved the AIC by two points; however, this resulted in reduced overall model performance, and hence age was retained in the final model. In contrast, sex was removed from the survival model during backward stepwise selection, as its exclusion improved the AIC without materially affecting model performance. Adjusted odds ratios for all terms in both final models are reported in *Table 3*. Full model performance metrics on the development dataset are summarized in *Table 4*, with ROC curves displayed in *Figure 1*. The ROSC model had a ROC AUC of 0.702 [95% confidence interval (CI): 0.694–0.711] and Brier score of 0.182. The survival model performed better with a ROC AUC of 0.877 (95% CI: 0.868–0.887) and Brier score of 0.059.

**Table 3:** Multivariable models adjusted odds ratios

Variable	ROSC at hospital handover odds ratio (95% CI)	Survival to hospital discharge odds ratio (95% CI)
Age	1.000 (0.998–1.002)	0.970 (0.967–0.974)
Sex		
Male	1	N/A
Female	1.319 (1.226–1.420)	
Witness/bystander CPR		
Unwitnessed and no bystander CPR	1	1
Unwitnessed and bystander CPR provided	0.898 (0.784–1.028)	0.775 (0.568–1.063)
Bystander witnessed and no bystander CPR	1.408 (1.220–1.625)	1.449 (1.069–1.976)
Bystander witnessed and bystander CPR provided	1.603 (1.420–1.812)	1.708 (1.321–2.234)
EMS witnessed	1.739 (1.520–1.991)	4.427 (3.389–5.848)
Aetiology		
Medical	1	1
Asphyxia	2.007 (1.634–2.457)	1.089 (0.623–1.785)
Drowning	0.677 (0.294–1.362)	0.915 (0.144–3.194)
Overdose	1.571 (1.202–2.037)	2.366 (1.530–3.562)
Other (non-cardiac)	1.187 (1.051–1.338)	0.571 (0.432–0.745)
Traumatic and exsanguination	0.839 (0.651–1.070)	0.219 (0.097–0.429)
Initial rhythm		
Shockable (VF/VT)	1	1
Asystole	0.212 (0.194–0.231)	0.042 (0.034–0.050)
PEA	0.392 (0.357–0.431)	0.101 (0.085–0.120)

CPR, cardiopulmonary resuscitation; EMS, emergency medical services; PEA, pulseless electrical activity; ROSC, return of spontaneous circulation; VF, ventricular fibrillation; VT, ventricular tachycardia.

**Table 4:** Model performance on development and validation datasets ROSC: return of spontaneous circulation

	ROSC at hospital handover		Survival to hospital discharge	
	Development dataset *n* = 17 709	Validation dataset *n* = 20 722	Development dataset *n* = 17 546	Validation dataset *n* = 20 143
ROC AUC	0.702 (0.694–0.711)	0.712 (0.704–0.719)	0.877 (0.868–0.887)	0.871 (0.862–0.879)
Brier score	0.182	0.182	0.059	0.061
Hosmer–Lemeshow test *P*-value	<0.001	< 0.001	< 0.001	< 0.001
Accuracy	0.734	0.735	0.921	0.917
Sensitivity	0.897	0.897	0.990	0.991
Specificity	0.323	0.334	0.179	0.145
Positive predictive value	0.770	0.769	0.929	0.924
Negative predictive value	0.553	0.567	0.617	0.614
Akaike Information Criterion	19 280	N/A	7154	N/A

ROC AUC (area under the receiver operating characteristic curve): Measures the model’s ability to discriminate between classes. Values closer to 1 indicate better performance. Brier score: accuracy of probabilistic predictions, incorporating calibration and discrimination; lower scores indicate better performance. Hosmer–Lemeshow test: Assesses logistic regression goodness-of-fit Accuracy: Proportion of all cases correctly classified. Sensitivity: proportion of true positives correctly identified. Specificity: proportion of true negatives correctly identified. Positive predictive value: proportion of predicted positives that are true positives. Negative predictive value: proportion of predicted negatives that are true negatives. Akaike Information Criterion: A measure of model quality that balances goodness-of-fit with model complexity. Lower values indicate better models.

### Model validation

**Figure 2:**
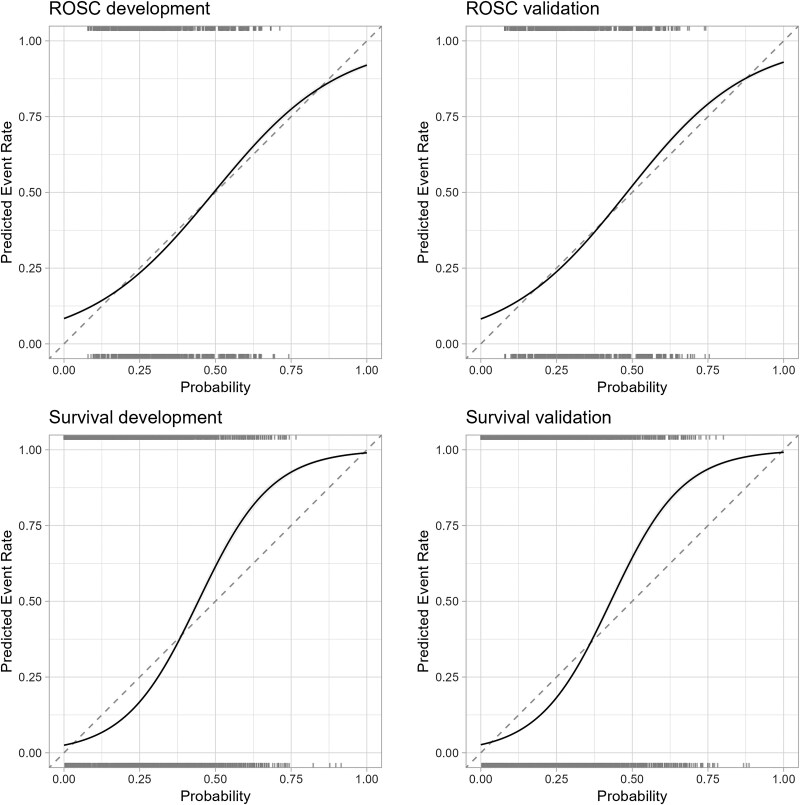
Calibration plots. ROSC: Return of spontaneous circulation.

Validation was conducted using complete cases only, comprising 20 722 cases for the ROSC model and 20 143 for the survival to hospital discharge model. Full model performance metrics on the validation dataset are summarized in *Table 4*, with ROC curves illustrated in *Figure 1*. Both models demonstrated consistent performance across development and validation datasets, with similar ROC AUC and classification accuracy. Brier scores indicated good performance in both datasets. As in the development dataset, the survival model performed better than the ROSC model, with survival model ROC AUC of 0.871 (95% CI: 0.862–0.879) and Brier score of 0.061, compared with ROSC model ROC AUC of 0.712 (95% CI: 0.704–0.719) and Brier score of 0.182. However, calibration for the survival model declined at higher predicted probabilities of survival, where survival was over-predicted (*Figure 2*). The same sigmoidal pattern was present in calibration plots for ROSC model but at a lower magnitude than the survival model.

### Sensitivity analysis

Public access defibrillator use had high missingness (43.6% across all data). Its inclusion in the ROSC model gave a development dataset of 14 554 cases and a validation dataset of 14 713 cases, and for survival produced a development dataset of 14 437 cases and a validation dataset of 14 448 cases. Its inclusion in these final models did not materially alter model performance (see Supplementary Material). Further sensitivity analyses were conducted removing potentially influential outliers. In the ROSC model, eight potential outliers were excluded, resulting in near identical model performance with no impact on AUC. In the survival model, two potential outliers excluded, again with near identical model performance and no impact on AUC (see Supplementary Material). Aetiology was recoded to combine asphyxia, drowning, and overdose into a single category as previous models.^25^ This recoding produced immaterial change in the performance of either model (see Supplementary Material). A sensitivity analysis reintroducing sex into the survival model after its removal by backward stepwise selection produced almost identical ORs and confidence intervals for all variables, alongside almost identical model performance metrics (see Supplementary Material). Multiple imputation for missing data in the validation datasets produced pooled results that were almost identical to the complete case analysis (see Supplementary Material).

## Discussion

This study has developed and validated risk-adjusted models for ROSC at hospital handover and survival to hospital discharge following OHCA, using a large national registry dataset. The overall rate of ROSC was 28.5% and of survival was 8.2%. Aetiology, initial rhythm, and witnessed/bystander CPR status were the most influential predictors of these outcomes. Both models demonstrated strong performance, with the survival model showing excellent discrimination and calibration. However, calibration plots indicated that the survival model tended to overestimate survival at higher predicted probabilities, suggesting caution is warranted near the upper end of the probability range. Model performance was consistent across development and validation datasets, suggesting these models are robust and generalizable across time periods, strengthening their potential utility. Sensitivity analyses confirmed the stability of model performance, enhancing the reliability of findings.

A comparison of previous and current models is included in *Table 5*, highlighting differences in population, outcome measures, statistical methods, predictors, and model performance. This study’s models have demonstrated improved performance and have a simpler structure compared with previous models using the OHCAO registry, likely reflecting improvements in data quality and full integration of the registry across English ambulance services.^25^ This study differs from Ji *et al.* by not using interaction terms and focusing on fixed-effects models to improve interpretability. The ROSC after cardiac arrest (RACA) and Utstein-Based ROSC (UB-ROSC) scores were both developed using smaller sampler and similar variables, with the addition of Utstein location, EMS response time, and dichotomized age at 80 years.^32,33^ A key difference with RACA was the definition of ROSC, which was any ROSC defined as a palpable pulse for >20 s. This study’s ROSC model performed comparably to RACA but was outperformed by UB-ROSC (AUC of 0.83 in development and 0.77 in validation), which was based on regional data from Northern Italy and Southern Switzerland. The use of national registry data in the current study may improve generalizability compared with regional datasets. Despite strong model performance, there remains scope to improve calibration at high predicted survival probabilities and prediction of ROSC. This study used a traditional statistical approach with historic data, a potential limitation. Machine learning models have achieved high performance in predicting survival, with AUC as high as 0.90 to 0.97.^34-37^ However, these often rely on a larger set of predictors, including emergency department and in-hospital variables, which may limit their clinical utility. Living models, which are continuously updated with new registry data, may offer an elegant solution to maintaining performance over time and could be integrated with machine learning approaches.^38,39^ Future research should explore how machine learning combined with live registry data could produce continually updated, accurate, and interpretable models to inform clinicians and policymakers.

**Table 5:** Comparison of similar models in the current published literature

Name	Setting and population	Outcome	Statistical approach and final model variables	ROC AUC (development)	ROC AUC (validation)
**ROSC**					
Current study	National: England (Nine ambulance services) All OHCA. Development *n* = 17 709 (2016) Validation *n* = 20 722 (2017)	ROSC to hospital handover	Traditional regression. All fixed effects. Age, sex, witnessed/bystander CPR status, aetiology, initial rhythm	0.70 (95% CI 0.69–0.71)	0.71 (95% CI 0.70–0.72)
Ji^25^	National: England (Seven ambulance services) All OHCA Development *n* = 16 470 (2014) Validation *n* = 16 319 (2015)	ROSC to hospital handover	Traditional regression. Mixed effects model. Fixed effects: gender, witnessed/bystander CPR, aetiology, initial rhythm. Interactions: witness/bystander CPR & gender, witnessed/bystander CPR & aetiology, witnessed/bystander CPR & initial rhythm, aetiology &initial rhythm	0.70 (95% CI 0.69–0.71)	0.67 (95% CI 0.66–0.68)
UB-ROSC^33^	Regional: One region of Italy and one region of Switzerland All OHCA Development *n* = 1962 (2015–2017) Validation *n* = 747 (2018)	ROSC to hospital handover	Traditional regression. Mixed effects model. Sex, age, aetiology, location, witnessed/bystander CPR status, initial rhythm, time to EMS arrival.	0.83 (95% CI 0.81–0.85)	0.77 (95% CI 0.74–0.80)
RACA^32^	National: Germany All OHCA Development *n* = 5471 (1998 to 2008) Validation *n* = 2218 (2009–2010)	Any ROSC (palpable pulse for >20 s)	Traditional regression. All fixed effects. Sex, age, aetiology, witnessed status, location, initial rhythm, bystander CPR, time to EMS arrival.	0.71 (95% CI 0.70–0.72).	0.731 (95% CI 0.71–0.75)
**Survival**					
Current study	National: England (Nine ambulance services) All OHCA. Development *n* = 17 546 (2016) Validation *n* = 20 143 (2017)	Survival to hospital discharge	Traditional regression. All fixed effects. Age, witnessed/bystander CPR status, aetiology, initial rhythm	0.88 (95% CI 0.870–0.89)	0.87 (95% CI 0.86–0.88)
Ji^25^	National: England (seven ambulance services) All OHCA Development *n* = 10 648 (2014) Validation *n* = 13 686 (2015)	Survival to hospital discharge	Traditional regression. Mixed effects model. Fixed effects: age, gender, witnessed/bystander CPR, aetiology, initial rhythm. Interactions: witnessed/bystander CPR & gender, witnessed/bystander CPR & aetiology, witnessed/bystander CPR & initial rhythm, aetiology &initial rhythm	0.85 (95% CI 0.84–0.86)	0.87 (95% CI 0.86–0.88)
Kwon^34^	National: Korea All OHCA with sustained ROSC (>20 min) Development *n* = 28 045 (2012–2015) Validation *n* = 8145 (2016)	Survival to hospital discharge	Deep learning (multilayer perceptron). Also constructed machine learning models with logistic regression (LR), support vector machine (SVM), and random forest (RF). Age, sex, location, aetiology, ROSC before ED, witnessed status, bystander CPR, initial rhythm with EMS, initial rhythm in ED, time from ED visit to ROSC	Deep learning: 0.93 LR: 0.89 SVM: 0.86 RF: 0.89	Deep learning: 0.90 (95% CI 0.90–0.90) LR: 0.89 (95% CI 0.88–0.89) SVM: 0.84 (95% CI 0.83–0.84) RF: 0.87 (95% CI 0.87–0.88)
SCARS-1^35^	National: Sweden All OHCA Training *n* = 33 370 (2010–2020) Evaluation *n* = 11 123 (2010–2020) Test *n* = 11 122 (2010–2020)	30-day survival	Machine learning (extreme gradient boosting) 393 candidate predictors	Evaluation data: 0.975	Test data: 0.97
SCARS-2^36^	National: Sweden All OHCA Training *n* = 33 370 (2010–2020) Evaluation *n* = 11 123 (2010–2020) Test *n* = 11 122 (2010–2020)	30-day survival	Machine learning [extreme gradient boosting (XGB), LightGBM] 384 candidate predictors, with top 40 predictors used.	Not reported	Test data: XGB: 0.96. LightGBM: 0.96.
Seki^37^	Regional: Kanto area of Japan Adult OHCA with presumed cardiac aetiology. Development *n* = 5718 (2012) Validation *n* = 1608 (2013)	Survival at 1-year	Machine learning (random forest) Model with 35 variables (all prehospital) and model with 53 variables (35 prehospital and 18 in-hospital)	Not reported	Prehospital variables only (*n* = 35): 0.94 (95% CI 0.93–0.96) Prehospital and in-hospital variables (*n* = 53): 0.96 (95% CI 0.95–0.97)

CI, confidence interval; CPR, cardiopulmonary resuscitation; OHCA, out-of-hospital cardiac arrest; ROC AUC, area under the receiver operating characteristic curve; ROSC, return of spontaneous circulation.

The models developed in this study may be best suited to system-level applications, such as supporting quality improvement, benchmarking, and guiding resource allocation. By adjusting for case mix and differences in patient characteristics, benchmarking can determine whether observed survival rates reflect expected outcomes. This approach enables identification of underperforming areas, allowing further investigation to understand patterns and better direct quality improvement, whilst also highlighting areas of excellence to share best practice and lessons learned.^40,41^ These models may also support quality improvement by providing an evidence-based framework to monitor progress over time, using outcomes standardized to patients’ presenting OHCA characteristics. By distinguishing patient-related risk from system-level factors, the models can support a data-driven approach to equitable resource allocation and targeted interventions.

This study identifies the key predictors of ROSC and survival following OHCA. Initial rhythm remains the strongest independent predictor of survival.^25,42^ Patients with shockable presenting rhythms had markedly improved survival compared to PEA or asystole. This may inform triage of specialist prehospital services and in-hospital decision-making around burdensome and advanced therapies, including extracorporeal CPR.^43-46^ Witnessed arrest, particularly EMS-witnessed, alongside the provision of bystander CPR were also strong determinants of survival, emphasizing the critical role of early recognition and community response.^47-49^ Age was inversely associated with survival but did not predict ROSC. The identification of modifiable factors such as bystander CPR, and non-modifiable factors such as age can inform both immediate clinical priorities, as well as broader public health strategies. At the individual patient level, these predictors may be used by prehospital and in-hospital practitioners to inform decision-making, including around appropriateness of prolonged and aggressive resuscitation, prioritization of advanced interventions, and prognostic decisions. A strength of this study is its multi-centre and national setting, which improves generalizability compared with single-centre prediction models used in clinical practice for comatose post-OHCA patients.^50-52^ Nevertheless, while predictive models may offer objectivity, patients and society may be uncomfortable with over-reliance on mathematical models, instead favouring their role as a supplement to inform personalized, collaborative shared decision-making by expert clinicians.^53-57^

This study has several limitations. First, the models were developed and validated using data from England and therefore may reflect national or system-level characteristics that limit generalisability to other healthcare settings. Second, the final multivariable models were based on complete case analysis, and variables with >10% missingness in the development dataset included age, witnessed/bystander CPR, and aetiology. Although this approach avoided imputation bias, it substantially reduced the sample size and may introduce bias if missingness was not random. Sensitivity analyses using multiple imputation for validation produced similar performance, providing some reassurance about the robustness of models. Third, residual or unmeasured confounding may persist, particularly if there are important variables not captured within the OHCAO registry. Nevertheless, the registry follows the internationally recognized Utstein template, which provides standardization.^24^ Similarly, despite quality assurance processes, the use of registry-based data still carries a risk of misclassification bias. Fourth, the data used was from 2016 and 2017. Although the models performed well on temporally separate validation data, clinical practice and OHCA epidemiology may have changed, including due to public health initiatives.^58,59^ In particular, there may be impacts during and after the COVID-19 pandemic. Future work should consider validating these models using more recent data to confirm their applicability. Fifth, some subgroups, such as special circumstance arrests, had low frequencies and have distinct cardiac arrest physiology that may not be captured by the model. Sixth, survival was measured at hospital discharge, which may introduce variability as length of hospital stay can differ across subgroups, for example, patients with overdose or other reversible causes which may recover more quickly. Length of hospital stay was not captured, limiting exploration of these nuances. The registry now collects survival at 30 days, which may provide a more consistent endpoint for future validation studies. Nevertheless, this impact may be limited as previous work has shown survival to hospital discharge and 30 days is similar.^60^ Survival with a favourable neurological outcome may also be a more patient-centred outcome, and future studies should consider involving patients and the public in study design to ensure research addresses their priorities. Finally, had the dataset been randomly split into training and testing subsets rather than by year, differences in model parameters may have arisen if there were changes between years. We used distinct calendar years for development and validation, therefore allowing temporal external validation and providing an improved assessment of model stability over time and enhancing generalizability to future years.

## Conclusion

This study has developed and validated risk adjustment models for ROSC and survival to hospital discharge following OHCA using routinely collected national registry data. The models performed well in both development and validation datasets and offer a transparent, evidence-based approach to risk adjustment in OHCA. Future work should explore how these models can support comparative reporting, quality improvement, and if more complex modelling approaches, for example, machine learning, could yield improved model performance.

## Supplementary Material

qcaf159_Supplementary_Data

## Data Availability

Requests for access to aggregated data should be addressed to the corresponding author or to the data custodian.
