# Supplementary material for: Development and validation of multivariable risk adjustment models for return of spontaneous circulation and survival to hospital discharge following out-of-hospital cardiac arrest in England
Source: Eur Heart J Qual Care Clin Outcomes. 2025 Dec 18;12(4):439–50. doi: 10.1093/ehjqcco/qcaf159 (PMC13288742; doi:10.1093/ehjqcco/qcaf159)
Supplement: qcaf159_Supplementary_Data [file qcaf159_supplementary_data.docx]

Development and validation of multivariable prediction models for return of spontaneous circulation and survival following out-of-hospital cardiac arrest in England

Supplementary materials

Contents

[Missing data 2](#_Toc215056725)

[Derivation dataset description 2](#_Toc215056726)

[Validation dataset description 2](#_Toc215056727)

[Characteristics of included (complete cases) and excluded cases (incomplete cases) for ROSC datasets 3](#_Toc215056728)

[Pattern of missing data in ROSC derivation dataset 5](#_Toc215056729)

[Pattern of missing data in ROSC validation dataset 6](#_Toc215056730)

[Multiple imputation of the ROSC validation dataset 7](#_Toc215056731)

[Visualisations of imputed datasets 8](#_Toc215056732)

[Model performance on imputed validation datasets 10](#_Toc215056733)

[Characteristics of included (complete cases) and excluded cases (incomplete cases) from survival datasets 11](#_Toc215056734)

[Pattern of missing data in survival derivation dataset 13](#_Toc215056735)

[Pattern of missing data in survival validation dataset 14](#_Toc215056736)

[Multiple imputation of the survival validation dataset 15](#_Toc215056737)

[Visualisations of imputed datasets 16](#_Toc215056738)

[Model performance on imputed validation datasets 18](#_Toc215056739)

[Sensitivity analysis 19](#_Toc215056740)

[Potentially influential outliers 19](#_Toc215056741)

[ROSC model 19](#_Toc215056742)

[Survival model 20](#_Toc215056743)

[Survival model with sex: Effect estimates 21](#_Toc215056744)

[Sensitivity analysis model performance 22](#_Toc215056745)

[ROSC 22](#_Toc215056746)

[Survival 23](#_Toc215056747)

# Missing data

## Derivation dataset description

| **Variable** | **Missing count** | **Percentage for total dataset (%)** |
| --- | --- | --- |
| Age | 4042 | 14.5 |
| Sex | 466 | 1.7 |
| Witness/bystander CPR | 3977 | 14.2 |
| Aetiology | 2852 | 10.2 |
| Initial rhythm | 1774 | 6.4 |
| ROSC | 1559 | 5.6 |
| Survival | 1810 | 6.5 |

## Validation dataset description

| **Variable** | **Missing count** | **Percentage for total dataset (%)** |
| --- | --- | --- |
| Age | 3923 | 14.0 |
| Sex | 168 | 0.6 |
| Witness/bystander CPR | 2535 | 9.1 |
| Aetiology | 2263 | 8.1 |
| Initial rhythm | 1592 | 5.7 |
| ROSC | 1592 | 5.7 |
| Survival | 1775 | 6.4 |

## Characteristics of included (complete cases) and excluded cases (incomplete cases) for ROSC datasets

Percentages are calculated among cases with non-missing data for each characteristic to support comparisons.

| **Variable** | **Included development data (n=17,709)** | **Excluded development data (n=10,233)** | **Included validation data (n=20,722)** | **Excluded validation data (n=7,703)** |
| --- | --- | --- | --- | --- |
| **Age – median (IQR)** | 71 (25) | 72 (25) | 71 (24) | 72 (25) |
| Missing | 0 | 4042 | 0 | 2923 |
| **Sex – N (%)** |  |  |  |  |
| Male | 11284 (63.7) | 6267 (64.2) | 13206 (63.7) | 4872 (64.7) |
| Female | 6425 (36.3) | 3500 (35.8) | 7516 (36.3) | 2663 (35.3) |
| Missing | 0 | 466 | 0 | 168 |
| **Witnessed status – N (%)** |  |  |  |  |
| Unwitnessed | 6424 (36.3) | 3088 (44.8) | 7490 (36.1) | 2228 (41.2) |
| Bystander witnessed | 8301 (46.9) | 3049 (44.2) | 10402 (50.2) | 2557 (47.3) |
| EMS witnessed | 2984 (16.9) | 755 (11.0) | 2830 (13.7) | 625 (11.6) |
| Missing | 0 | 3341 | 0 | 2293 |
| **Bystander CPR – N (%)** |  |  |  |  |
| Yes | 10374 (58.6) | 4984 (62.2) | 12681 (61.2) | 3791 (60.5) |
| No | 7065 (39.9) | 2941 (36.7) | 7956 (38.4) | 2461 (39.3) |
| NA (EMS witnessed) | 270 (1.5) | 88 (1.1) | 85 (0.4) | 10 (0.2) |
| Missing | 0 | 2220 | 0 | 1441 |
| **Aetiology – N (%)** |  |  |  |  |
| Medical | 14758 (83.3) | 6408 (86.8) | 18054 (87.1) | 4614 (84.8) |
| Asphyxia | 500 (2.8) | 104 (1.4) | 663 (3.2) | 134 (2.5) |
| Drowning | 65 (0.4) | 22 (0.3) | 62 (0.3) | 28 (0.5) |
| Overdose | 323 (1.8) | 105 (1.4) | 364 (1.8) | 67 (1.2) |
| Traumatic | 432 (2.4) | 281 (3.8) | 521 (2.5) | 266 (4.9) |
| Exsanguination | 6 (0.0) | 4 (0.1) | 11 (0.1) | 2 (0.0) |
| Other (non-cardiac) | 1625 (9.2) | 457 (6.2) | 1047 (5.1) | 329 (6.0) |
| Missing | 0 | 2852 | 0 | 2263 |
| **Initial rhythm – N (%)** |  |  |  |  |
| Shockable (VF/VT) | 3993 (22.5) | 1539 (18.2) | 4704 (22.7) | 1156 (18.9) |
| Asystole | 9333 (52.7) | 5296 (62.6) | 11007 (53.1) | 3811 (62.4) |
| PEA | 4383 (24.8) | 1624 (19.2) | 5011 (24.2) | 1144 (18.7) |
| Missing | 0 | 1774 | 0 | 1592 |
| **ROSC at hospital handover – N (%)** |  |  |  |  |
| Yes | 5019 (28.3) | 2345 (27.0) | 5962 (28.8) | 1940 (30.9) |
| No | 12690 (71.7) | 6329 (73.0) | 14760 (71.2) | 4342 (69.1) |
| Missing | 0 | 1559 | 0 | 1421 |
| **Survival to hospital discharge – N (%)** |  |  |  |  |
| Yes | 1483 (8.5) | 556 (6.3) | 1758 (8.7) | 527 (8.1) |
| No | 15874 (91.5) | 8219 (93.7) | 18369 (91.3) | 5996 (91.9) |
| Missing | 0 | 1458 | 0 | 1180 |

## Pattern of missing data in ROSC derivation dataset


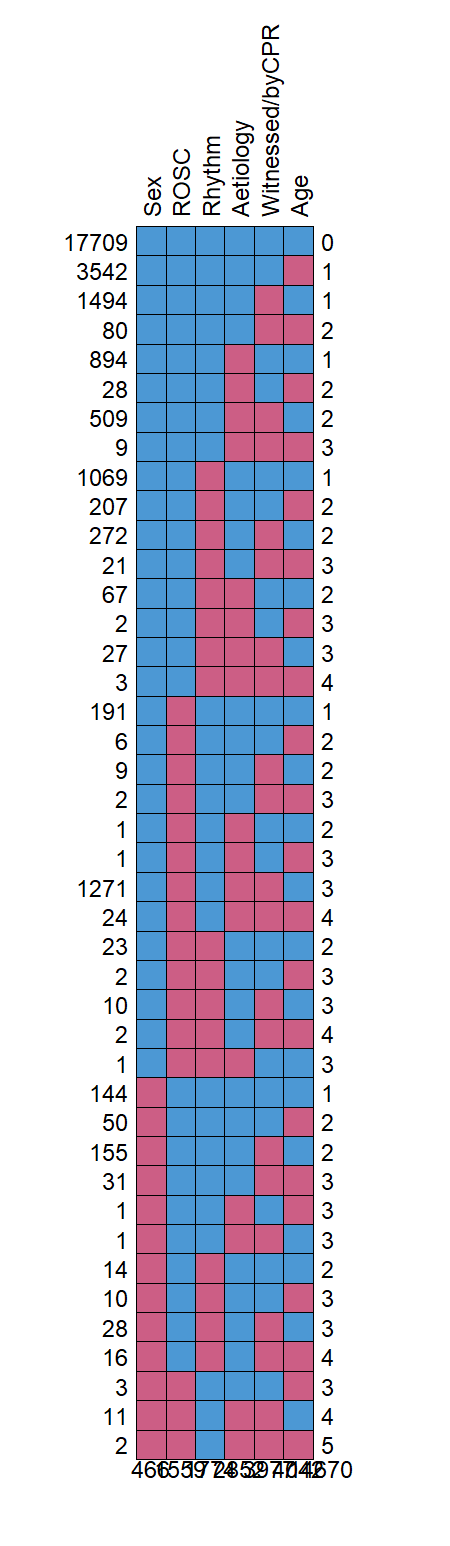


## Pattern of missing data in ROSC validation dataset


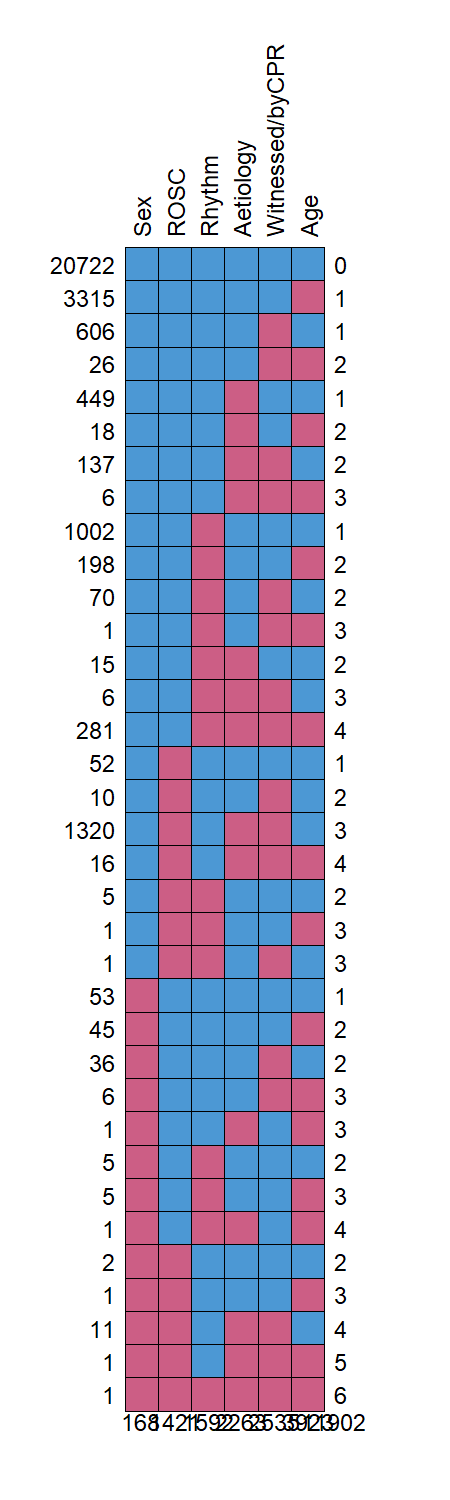


## Multiple imputation of the ROSC validation dataset

Twenty imputations of the missing data were performed by chain equations.

The following imputation methods were applied to each variable based on their data types:

- **Age**: Predictive Mean Matching (PMM)
- **Witness/bystander CPR status:** Polynomial Regression (PolyReg)
- **Aetiology**: Polynomial Regression (PolyReg)
- **Initial Rhythm**: Polynomial Regression (PolyReg)
- **ROSC**: No imputation

Below predictor matrix indicates the variables used in the imputation model for each variable:

| **Variable** | **Age** | **Witness/bystander CPR status** | **Aetiology** | **Initial rhythm** | **ROSC** |
| --- | --- | --- | --- | --- | --- |
| **Age** | 0 | 1 | 1 | 1 | 1 |
| **Witness/bystander CPR status** | 1 | 0 | 1 | 1 | 1 |
| **Aetiology** | 1 | 1 | 0 | 1 | 1 |
| **Initial rhythm** | 1 | 1 | 1 | 0 | 1 |
| **ROSC** | 1 | 1 | 1 | 1 | 0 |

A "0" indicates that the variable was not used as a predictor for imputation of that variable.

A "1" indicates that the variable was used as a predictor for imputation.

### Visualisations of imputed datasets


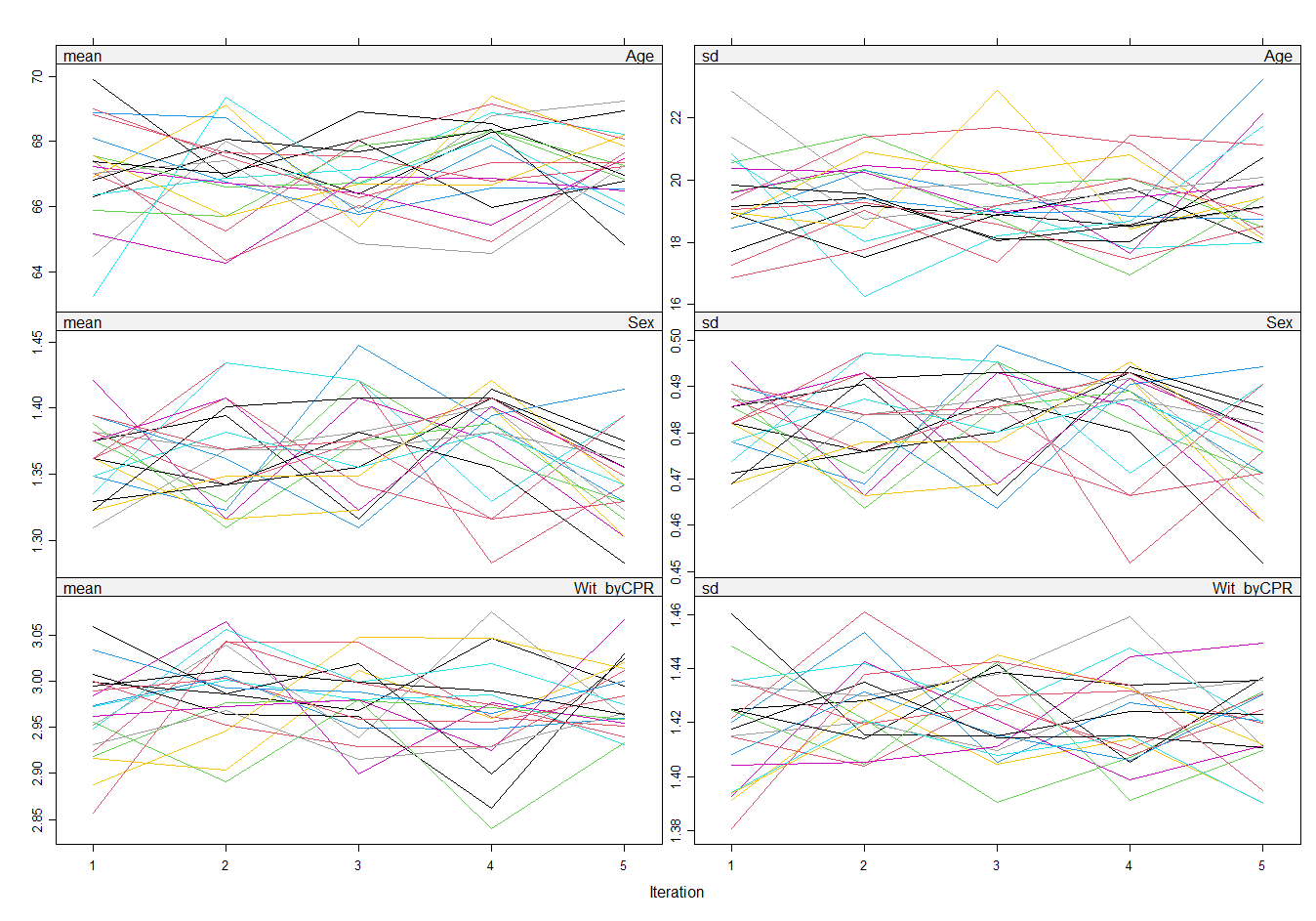


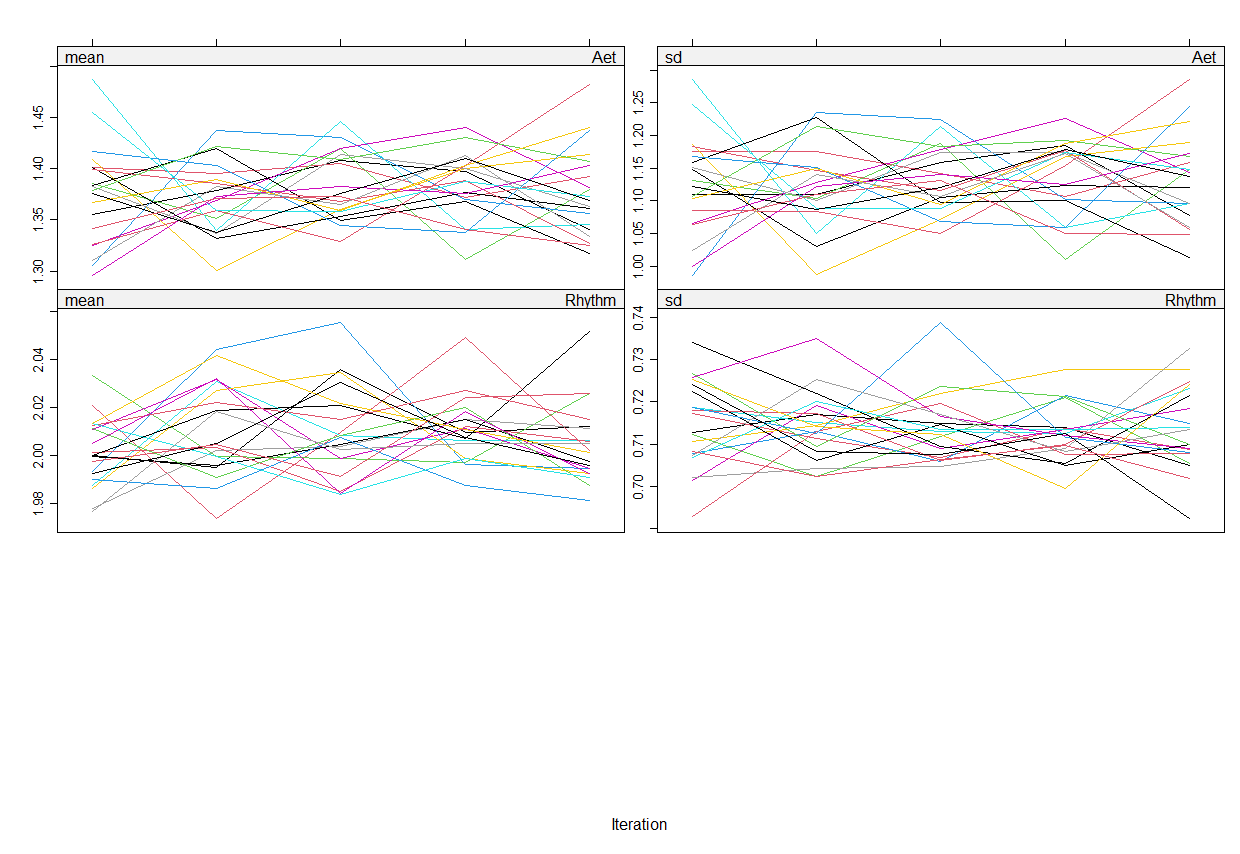


Each coloured line represents imputed dataset


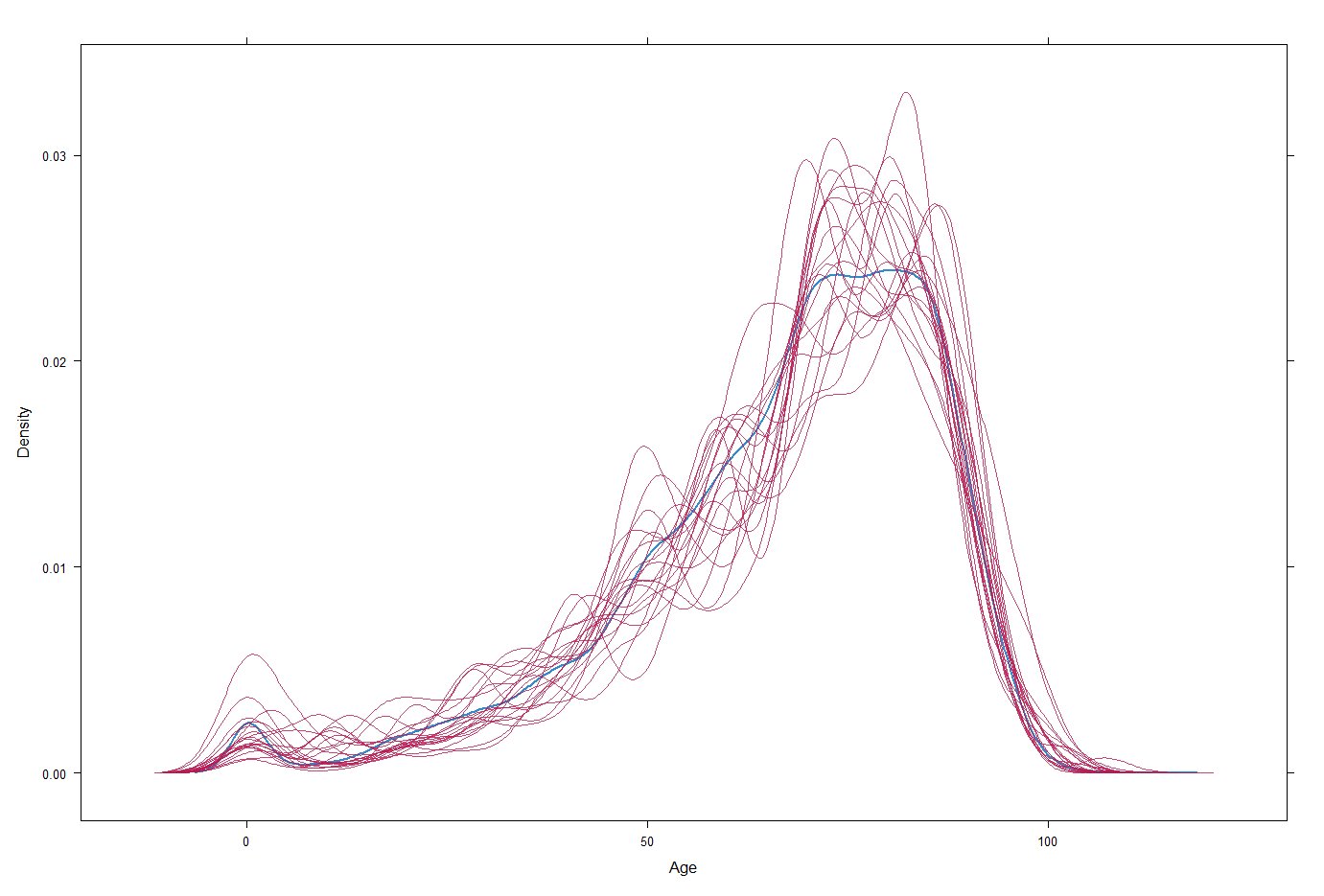


Blue line represents observed data, red lines represent imputed datasets

### Model performance on imputed validation datasets

|  | **Multiple imputation** |
| --- | --- |
|  | Pooled results across 20 imputed datasets (mean (SD)) |
| **ROC AUC (95% CI)** | 0.715 (0.709-0.722)  SD: 0.0009 (lower CI: 0.0009, upper CI: 0.0009)) |
| **Brier score** | 0.183 (0.0002) |
| **Hosmer–Lemeshow test p-value** | <0.001 |
| **Accuracy** | 0.733 (0.0004) |
| **Sensitivity** | 0.902 (0.0005) |
| **Specificity** | 0.327 (0.001) |
| **Positive predictive value** | 0.764 (0.0004) |
| **Negative predictive value** | 0.579 (0.001) |

## Characteristics of included (complete cases) and excluded cases (incomplete cases) from survival datasets

Percentages are calculated among cases with non-missing data for each characteristic to support comparisons.

| **Variable** | **Included development data (n=17,546)** | **Excluded development data (n=10,396)** | **Included validation data (n=20,143)** | **Excluded validation data (n=8,282)** |
| --- | --- | --- | --- | --- |
| **Age – median (IQR)** | 71 (24) | 72(25) | 71 (24) | 70 (26) |
| Missing | 0 | 4042 | 0 | 3923 |
| **Sex – N (%)** |  |  |  |  |
| Male | 11135 (63.5) | 6416 (64.6) | 12792 (63.5) | 5286 (65.1) |
| Female | 6411 (36.5) | 3514 (35.4) | 7351 (36.5) | 2828 (34.9) |
| Missing | 0 | 466 | 0 | 168 |
| **Witnessed status – N (%)** |  |  |  |  |
| Unwitnessed | 6396 (36.5) | 3116 (44.2) | 7326 (36.4) | 2392 (39.9) |
| Bystander witnessed | 8199 (46.7) | 3151 (44.7) | 10105 (50.2) | 2854 (47.7) |
| EMS witnessed | 2951 (16.8) | 788 (11.2) | 2712 (13.5) | 743 (12.4) |
| Missing | 0 | 3341 | 0 | 2293 |
| **Bystander CPR – N (%)** |  |  |  |  |
| Yes | 10283 (58.6) | 5075 (62.1) | 12342 (61.3) | 4130 (60.4) |
| No | 6991 (39.8) | 3015 (36.9) | 7729 (38.4) | 2688 (39.3) |
| NA (EMS witnessed) | 272 (1.6) | 86 (1.1) | 72 (0.4) | 23 (0.3) |
| Missing | 0 | 2220 | 0 | 1441 |
| **Aetiology – N (%)** |  |  |  |  |
| Medical | 14651 (83.5) | 6515 (86.4) | 17544 (87.1) | 5124 (85.1) |
| Asphyxia | 497 (2.8) | 107 (1.4) | 650 (3.2) | 147 (2.4) |
| Drowning | 63 (0.4) | 24 (0.3) | 59 (0.3) | 31 (0.5) |
| Overdose | 316 (1.8) | 112 (1.5) | 343 (1.7) | 88 (1.5) |
| Traumatic | 412 (2.3) | 301 (4.0) | 501 (2.5) | 286 (4.8) |
| Exsanguination | 6 (0.0) | 4 (0.1) | 8 (0.0) | 5 (0.1) |
| Other (non-cardiac) | 1601 (9.1) | 481 (6.4) | 1038 (5.2) | 338 (5.6) |
| Missing | 0 | 2852 | 0 | 2263 |
| **Initial rhythm – N (%)** |  |  |  |  |
| Shockable (VF/VT) | 3877 (22.1) | 1655 (19.2) | 4490 (22.3) | 1370 (20.5) |
| Asystole | 9333 (53.2) | 5296 (61.4) | 10799 (53.6) | 4019 (60.1) |
| PEA | 4336 (24.7) | 1671 (19.4) | 4854 (24.1) | 1301 (19.4) |
| Missing | 0 | 1774 | 0 | 1592 |
| **ROSC at hospital handover – N (%)** |  |  |  |  |
| Yes | 4828 (27.8) | 2536 (28.1) | 5643 (28.0) | 2259 (32.8) |
| No | 12529 (72.2) | 6490 (71.9) | 14484 (72.0) | 4618 (67.2) |
| Missing | 0 | 1370 | 0 | 1405 |
| **Survival to hospital discharge – N (%)** |  |  |  |  |
| Yes | 1485 (8.5) | 554 (6.5) | 1761 (8.7) | 524 (8.1) |
| No | 16061 (91.5) | 8032 (93.5) | 18382 (91.3) | 5983 (91.9) |
| Missing | 0 | 1810 | 0 | 1775 |

## Pattern of missing data in survival derivation dataset


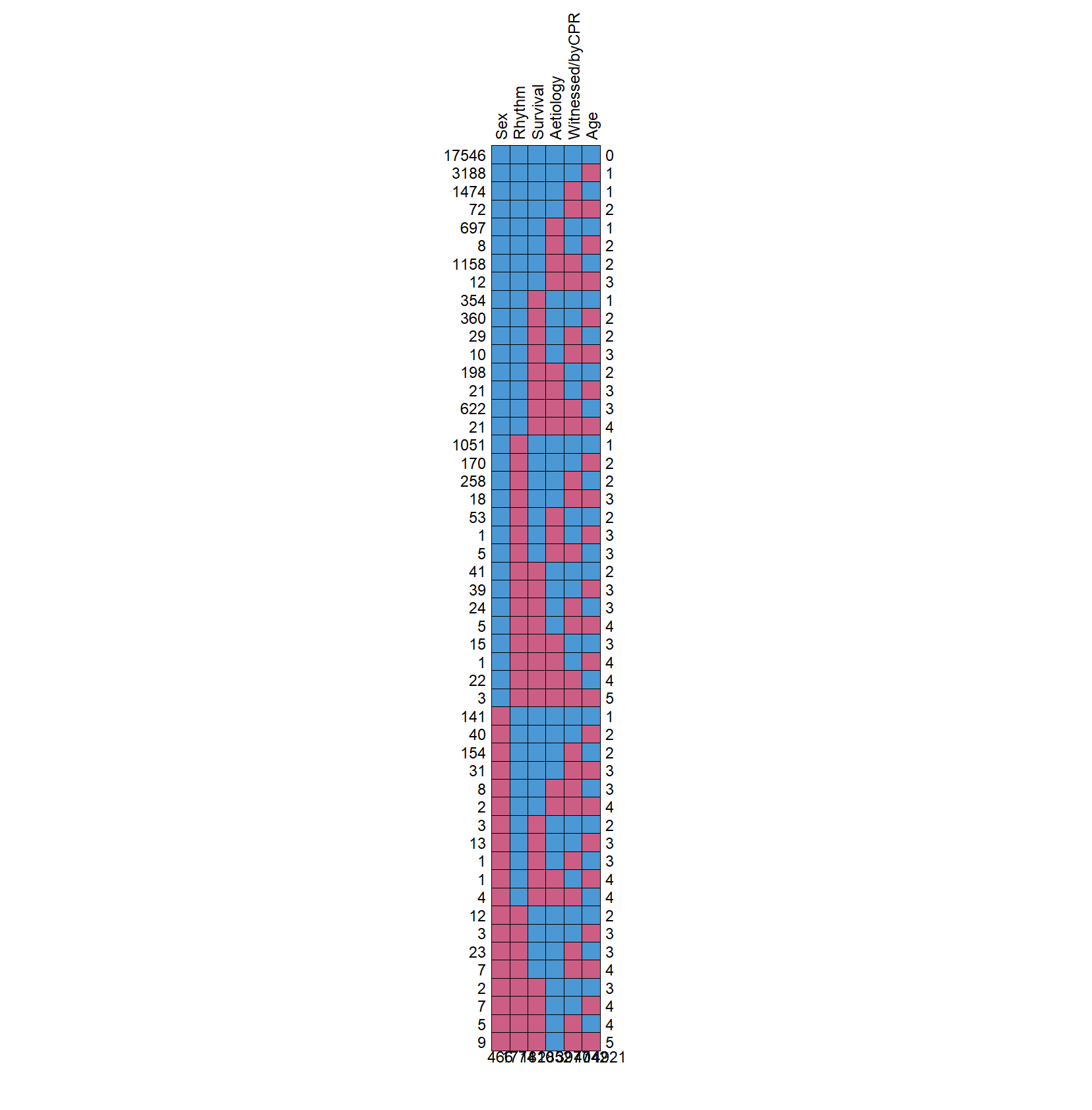


## Pattern of missing data in survival validation dataset


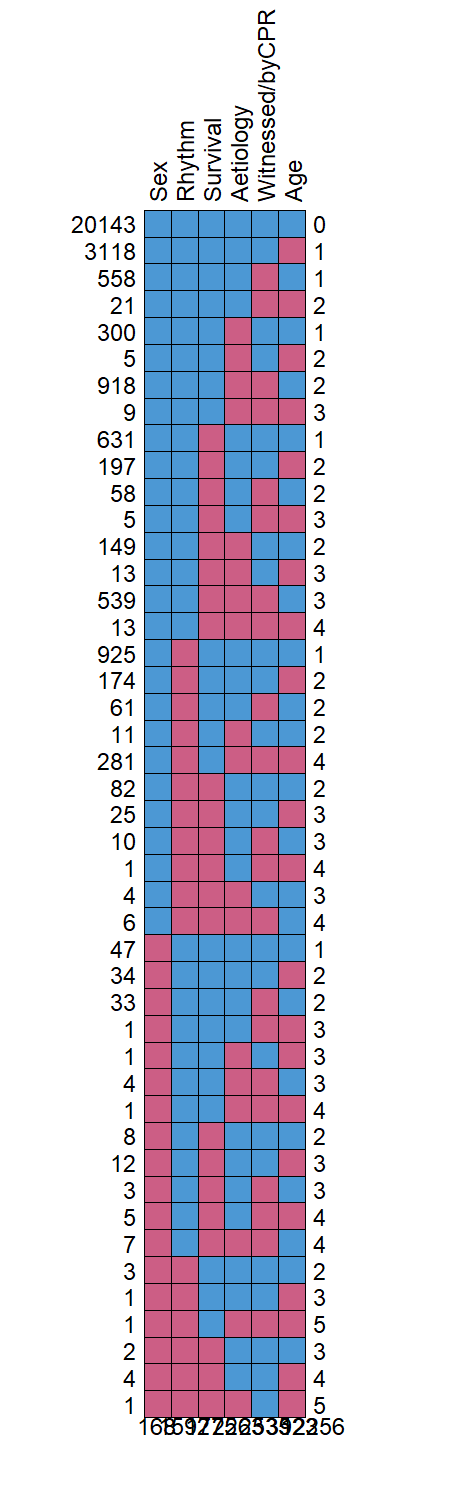


## Multiple imputation of the survival validation dataset

Twenty imputations of the missing data were performed by chain equations.

The following imputation methods were applied to each variable based on their data types:

- **Age**: Predictive Mean Matching (PMM)
- **Witness/bystander CPR status:** Polynomial Regression (PolyReg)
- **Aetiology**: Polynomial Regression (PolyReg)
- **Initial Rhythm**: Polynomial Regression (PolyReg)
- **Survival**: No imputation

Below predictor matrix indicates the variables used in the imputation model for each variable:

| **Variable** | **Age** | **Witness/bystander CPR status** | **Aetiology** | **Initial rhythm** | **Survival** |
| --- | --- | --- | --- | --- | --- |
| **Age** | 0 | 1 | 1 | 1 | 1 |
| **Witness/bystander CPR status** | 1 | 0 | 1 | 1 | 1 |
| **Aetiology** | 1 | 1 | 0 | 1 | 1 |
| **Initial rhythm** | 1 | 1 | 1 | 0 | 1 |
| **Survival** | 1 | 1 | 1 | 1 | 0 |

A "0" indicates that the variable was not used as a predictor for imputation of that variable.

A "1" indicates that the variable was used as a predictor for imputation.

### Visualisations of imputed datasets


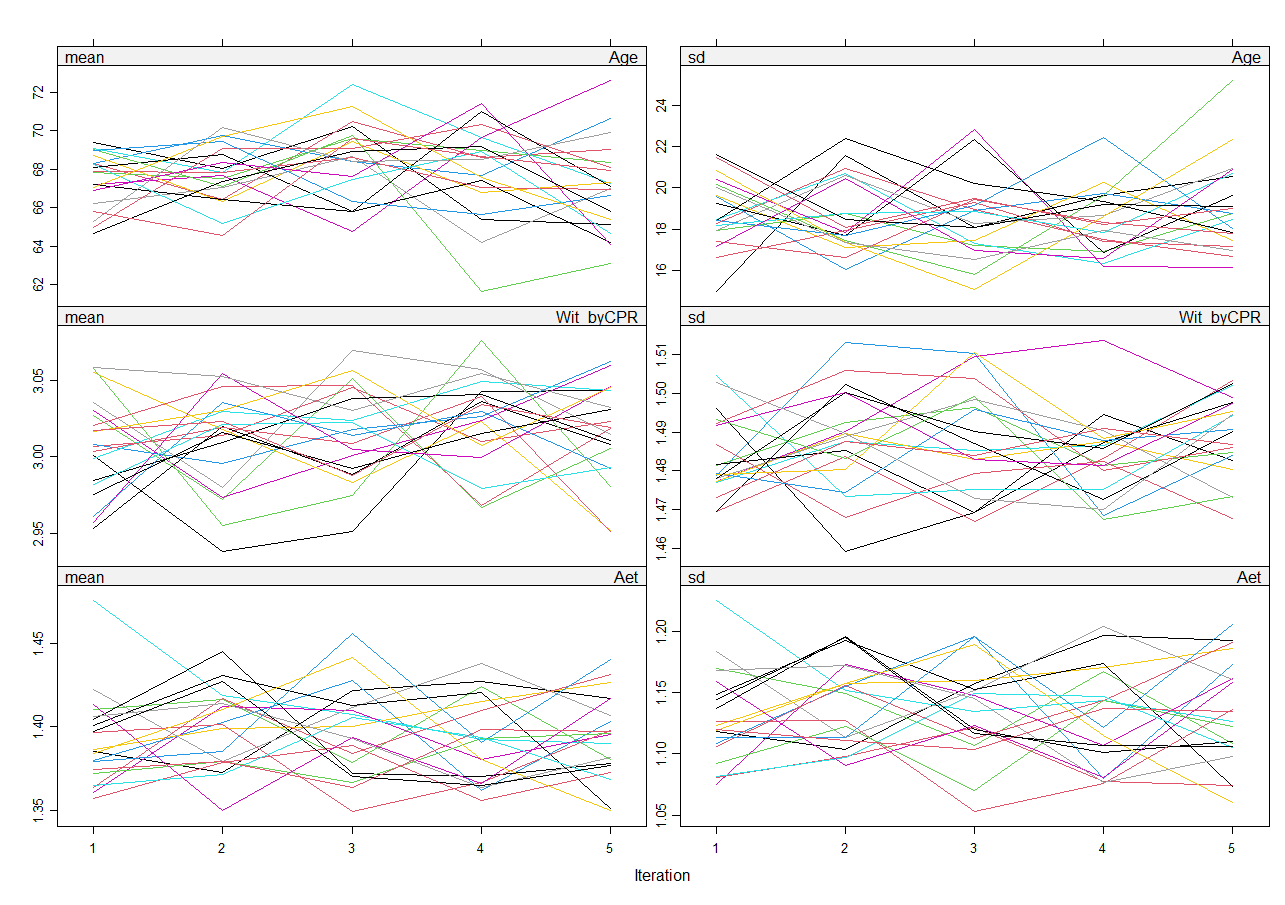


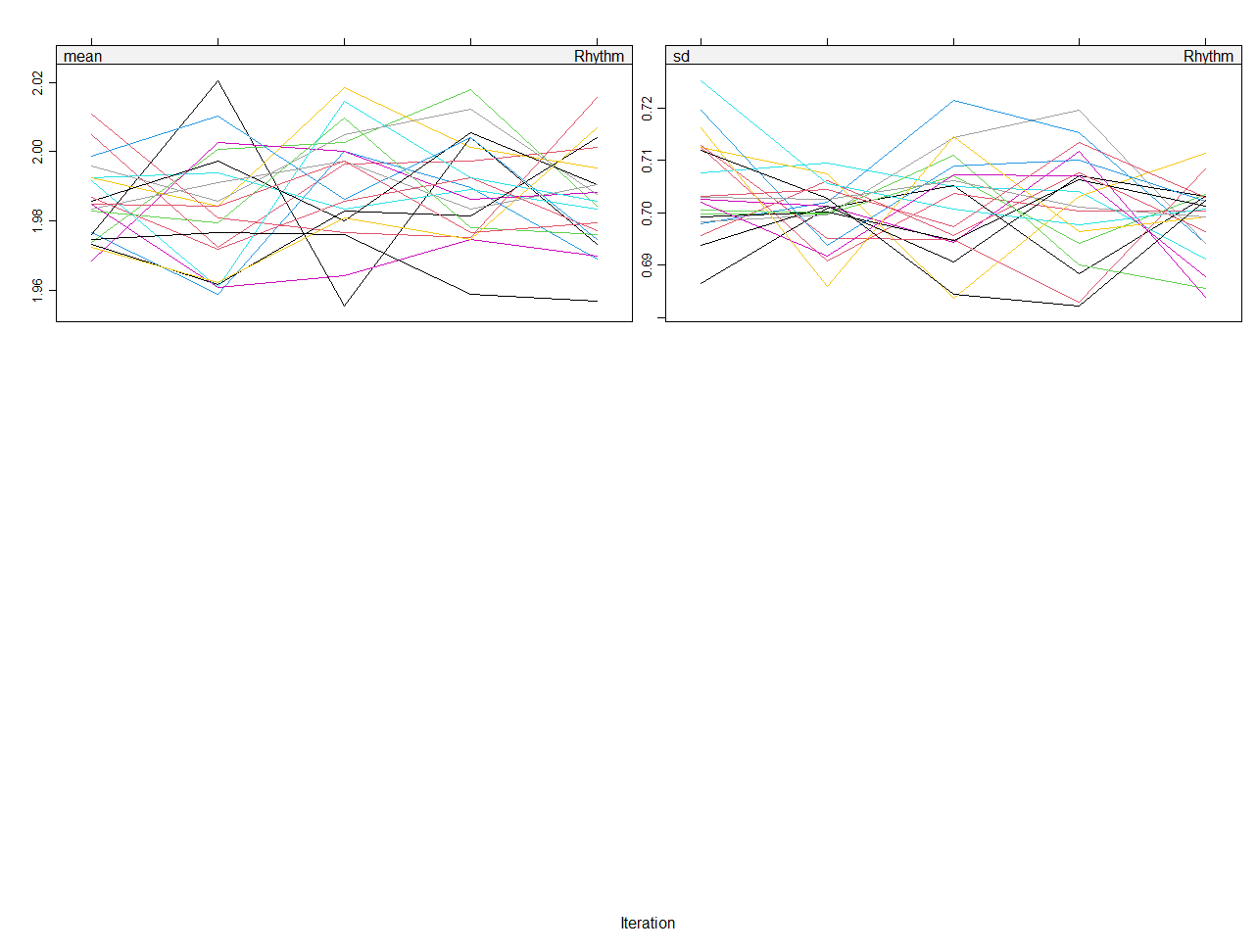


Each coloured line represents imputed dataset


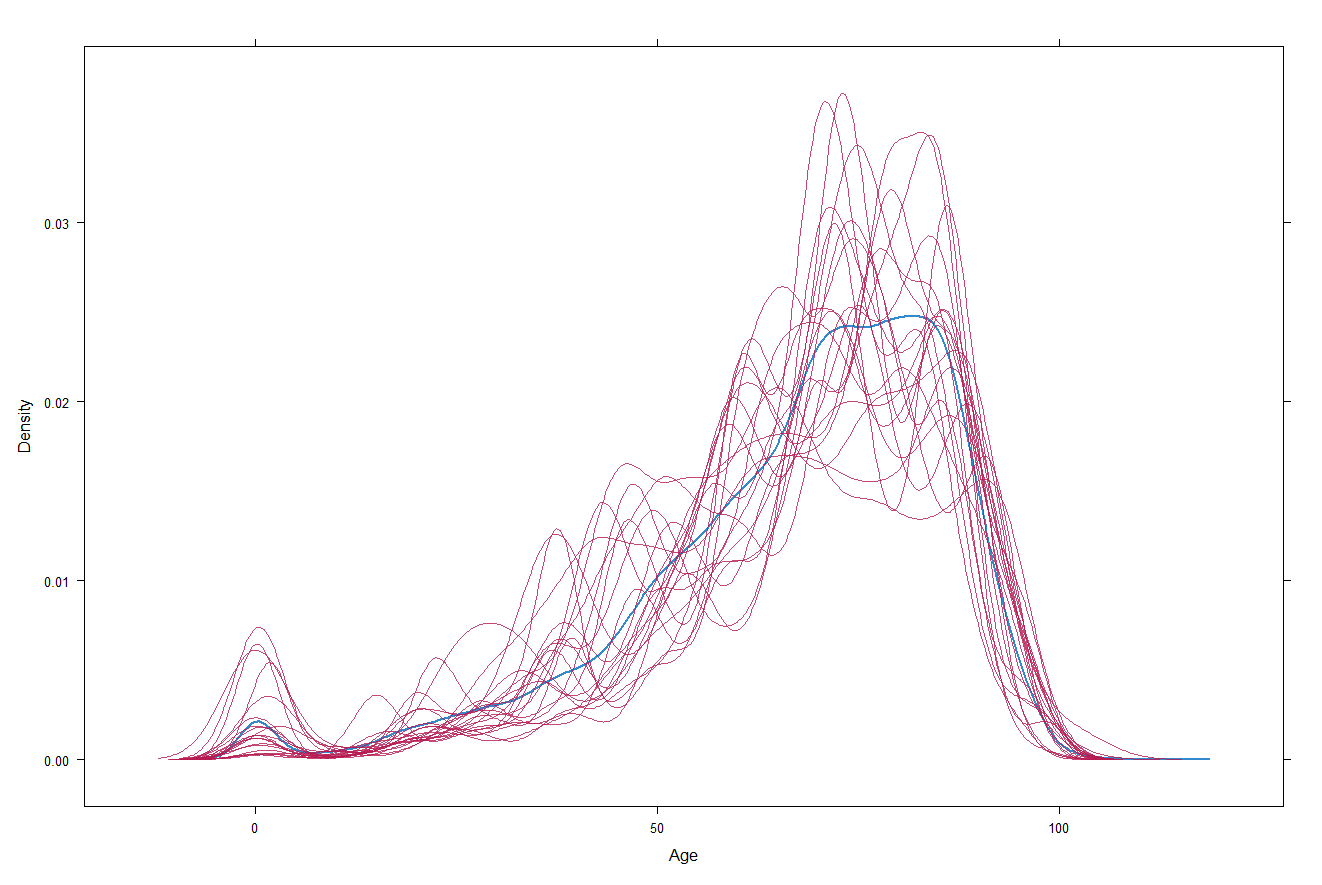


Blue line represents observed data, red lines represent imputed datasets

### Model performance on imputed validation datasets

|  | **Multiple imputation** |
| --- | --- |
|  | Pooled results across 20 imputed datasets (mean (SD)) |
| **ROC AUC (95% CI)** | 0.872 (0.864-0.880) (0.002 (0.002))  SD: 0.002 (lower CI: 0.002, upper CI: 0.002)) |
| **Brier score** | 0.062 (0.0004) |
| **Hosmer–Lemeshow test p-value** | <0.001 |
| **Accuracy** | 0.918 (0.0009) |
| **Sensitivity** | 0.991 (0.0007) |
| **Specificity** | 0.139 (0.006) |
| **Positive predictive value** | 0.925 (0.0005) |
| **Negative predictive value** | 0.605 (0.024) |

# Sensitivity analysis

## Potentially influential outliers

### ROSC model

Cook’s distance diagnostic plot for of all data:


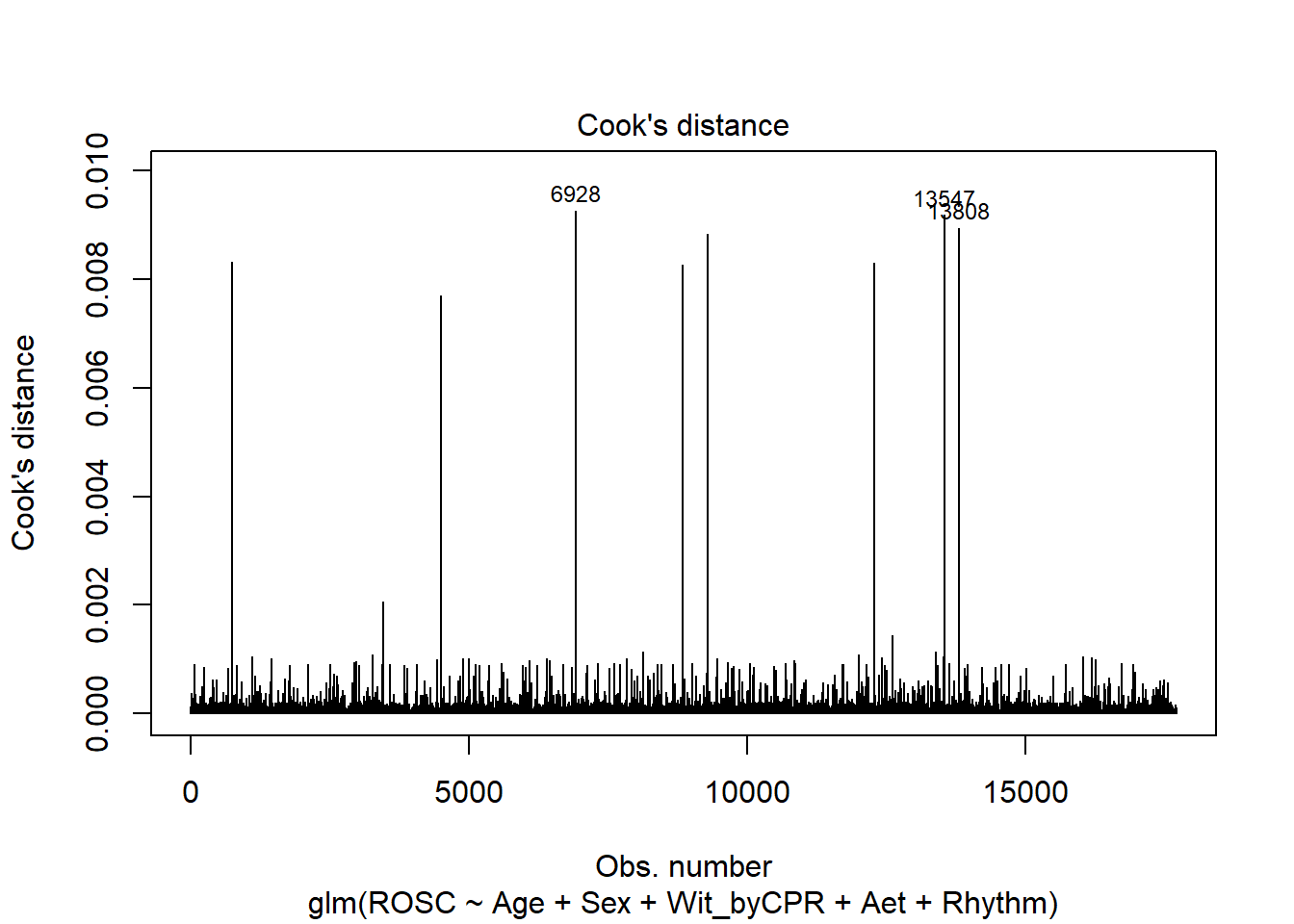


There are 8 observations of interest with much greater Cook's distance than the others, which were therefore removed.

### Survival model

Cook’s distance diagnostic plot for of all data:


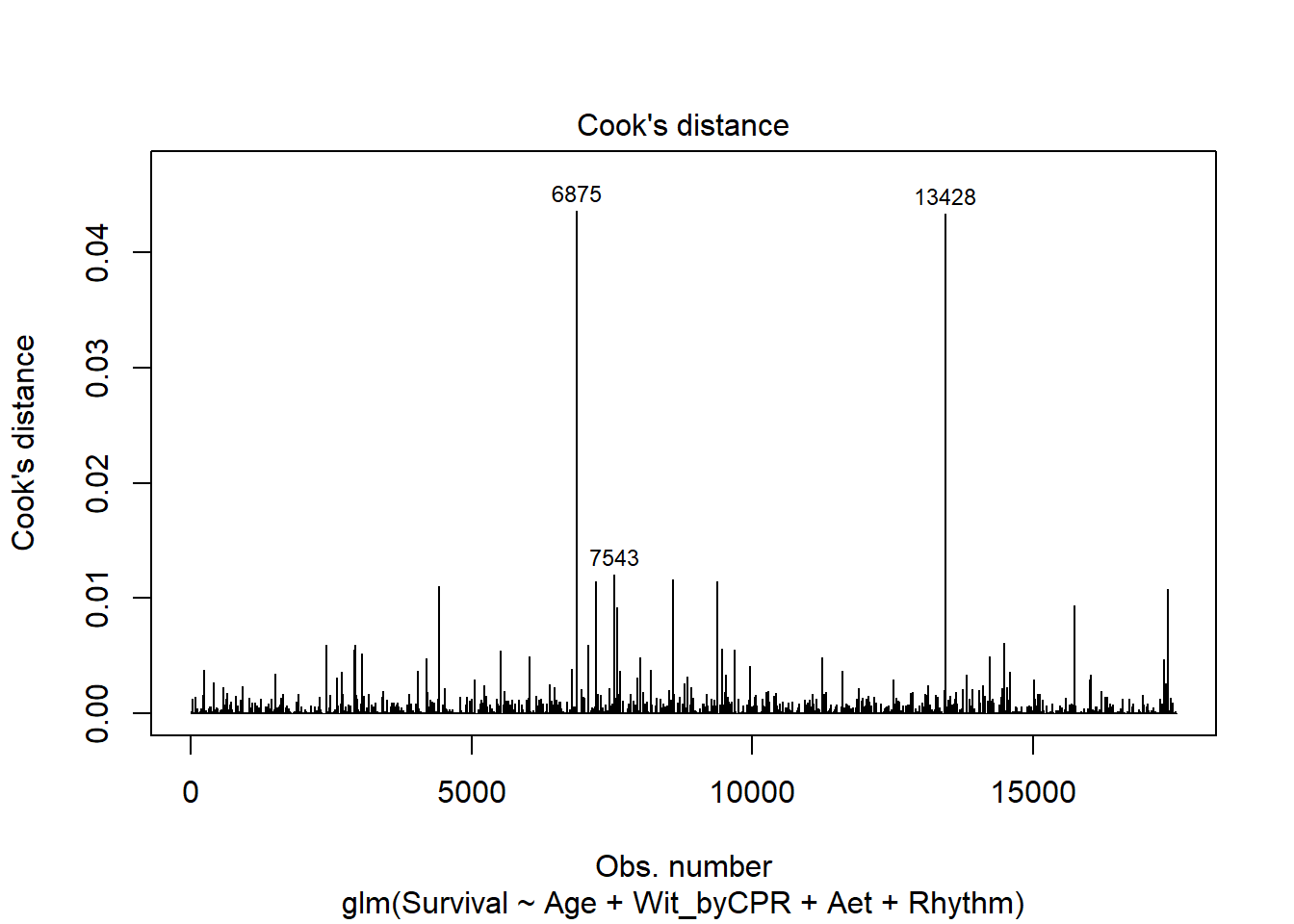


There are 2 observations of interest with much greater Cook's distance than the others, which were therefore removed.

# Survival model with sex: Effect estimates

| **Variable** | **Survival to hospital discharge**  **Odds ratio (95% confidence interval)** |
| --- | --- |
| Age | 0.970 (0.967-0.974) |
| Sex  Male  Female | 1  1.037 (0.967-1.189) |
| Witness/bystander CPR  Unwitnessed and no bystander CPR  Unwitnessed and bystander CPR provided  Bystander witnessed and no bystander CPR  Bystander witnessed and bystander CPR provided  EMS witnessed | 1  0.775 (0.568-1.063)  1.448 (1.069-1.975)  1.708 (1.321-2.234)  4.417 (3.381-5.835) |
| Aetiology  Medical  Asphyxia  Drowning  Overdose  Other (non-cardiac)  Traumatic and exsanguination | 1  1.089 (0.623-1.784)  0.916 (0.144-3.199)  2.366 (1.530-3.563)  0.569 (0.432-0.745)  0.222 (0.097-0.431) |
| Initial rhythm  Shockable (VF/VT)  Asystole  PEA | 1  0.042 (0.034-0.050)  0.100 (0.084-0.119) |

# Sensitivity analysis model performance

## ROSC

ROC AUC (Area Under the Receiver Operating Characteristic Curve): Measures the model's ability to discriminate between classes. Values closer to 1 indicate better performance. Brier score: accuracy of probabilistic predictions, incorporating calibration and discrimination; lower scores indicate better performance. Hosmer–Lemeshow test: Assesses logistic regression goodness-of-fit Accuracy: Proportion of all cases correctly classified. Sensitivity: proportion of true positives correctly identified. Specificity: proportion of true negatives correctly identified. Positive predictive value: proportion of predicted positives that are true positives. Negative predictive value: proportion of predicted negatives that are true negatives. AIC (Akaike Information Criterion): A measure of model quality that balances goodness-of-fit with model complexity. Lower values indicate better models.

|  | **ROSC at hospital handover** | | | | | |
| --- | --- | --- | --- | --- | --- | --- |
|  | **Public access defibrillator included** | | **Removal of potentially influential outliers** | | **Aetiology combination** | |
|  | Development dataset n=14,554 | Validation dataset n=14,713 | Development dataset n= 17,701 | Validation dataset n=20,722 | Development dataset n=17,709 | Validation dataset n=20,722 |
| **ROC AUC** | 0.692 (0.683-0.702) | 0.704 (0.695-0.713) | 0.702 (0.694-0.711) | 0.712 (0.704-0.719) | 0.701 (0.693-0.710) | 0.712 (0.704-0.719) |
| **Brier score** | 0.187 | 0.189 | 0.182 | 0.182 | 0.182 | 0.182 |
| **Hosmer–Lemeshow test p-value** | <0.001 | <0.001 | <0.001 | <0.001 | <0.001 | <0.001 |
| **Accuracy** | 0.724 | 0.723 | 0.734 | 0.735 | 0.734 | 0.734 |
| **Sensitivity** | 0.893 | 0.894 | 0.897 | 0.897 | 0.897 | 0.897 |
| **Specificity** | 0.317 | 0.330 | 0.323 | 0.334 | 0.323 | 0.331 |
| **Positive predictive value** | 0.758 | 0.754 | 0.770 | 0.769 | 0.770 | 0.768 |
| **Negative predictive value** | 0.553 | 0.577 | 0.553 | 0.567 | 0.553 | 0.565 |
| **AIC** | 16230 | N/A | 19230 | N/A | 19286 | N/A |

## Survival

ROC AUC (Area Under the Receiver Operating Characteristic Curve): Measures the model's ability to discriminate between classes. Values closer to 1 indicate better performance. Brier score: accuracy of probabilistic predictions, incorporating calibration and discrimination; lower scores indicate better performance. Hosmer–Lemeshow test: Assesses logistic regression goodness-of-fit Accuracy: Proportion of all cases correctly classified. Sensitivity: proportion of true positives correctly identified. Specificity: proportion of true negatives correctly identified. Positive predictive value: proportion of predicted positives that are true positives. Negative predictive value: proportion of predicted negatives that are true negatives. AIC (Akaike Information Criterion): A measure of model quality that balances goodness-of-fit with model complexity. Lower values indicate better models.

|  | **Survival to hospital discharge** | | | | | | | |
| --- | --- | --- | --- | --- | --- | --- | --- | --- |
|  | **Public access defibrillator included** | | **Removal of potentially influential outliers** | | **Aetiology combination** | | **Including sex** | |
|  | Development dataset n=14,437 | Validation dataset n=14,448 | Development dataset n=17,544 | Validation dataset n=20,143 | Development dataset n=17,546 | Validation dataset n=20,143 | Development dataset n=17,546 | Validation dataset n=20,143 |
| **ROC AUC** | 0.882 (0.872-0.891) | 0.870 (0.860-0.880) | 0.877 (0.868-0.886) | 0.870 (0.861-0.879) | 0.877 (0.867-0.886) | 0.870 (0.861-0.879) | 0.877 (0.868-0.887) | 0.870 (0.862-0.879) |
| **Brier score** | 0.059 | 0.063 | 0.058 | 0.061 | 0.059 | 0.061 | 0.059 | 0.061 |
| **Hosmer–Lemeshow test p-value** | <0.001 | <0.001 | <0.001 | <0.001 | <0.001 | <0.001 | <0.001 | <0.001 |
| **Accuracy** | 0.921 | 0.915 | 0.921 | 0.917 | 0.921 | 0.917 | 0.921 | 0.917 |
| **Sensitivity** | 0.989 | 0.989 | 0.989 | 0.991 | 0.989 | 0.91 | 0.989 | 0.991 |
| **Specificity** | 0.204 | 0.180 | 0.181 | 0.148 | 0.180 | 0.148 | 0.180 | 0.148 |
| **Positive predictive value** | 0.929 | 0.923 | 0.929 | 0.92 | 0.929 | 0.924 | 0.929 | 0.924 |
| **Negative predictive value** | 0.636 | 0.619 | 0.610 | 0.613 | 0.609 | 0.615 | 0.610 | 0.613 |
| **AIC** | 5908 | N/A | 7133 | N/A | 7158 | N/A | 7155 | N/A |
